# *SMN1* and *SMN2* copy numbers in cell lines derived from patients with spinal muscular atrophy as measured by array digital PCR

**DOI:** 10.1002/mgg3.141

**Published:** 2015-03-21

**Authors:** Deborah L Stabley, Ashlee W Harris, Jennifer Holbrook, Nicholas J Chubbs, Kevin W Lozo, Thomas O Crawford, Kathryn J Swoboda, Vicky L Funanage, Wenlan Wang, William Mackenzie, Mena Scavina, Katia Sol-Church, Matthew E R Butchbach

**Affiliations:** 1 Nemours Biomolecular Core Laboratory, Nemours Biomedical Research, Nemours Alfred I. duPont Hospital for ChildrenWilmington, Delaware; 2 Center for Applied Clinical Genomics, Nemours Biomedical Research, Nemours Alfred I. duPont Hospital for ChildrenWilmington, Delaware; 3 Department of Biological Sciences, University of DelawareNewark, Delaware; 4 Department of Neurology, Johns Hopkins UniversityBaltimore, Maryland; 5 Department of Pediatrics, Johns Hopkins UniversityBaltimore, Maryland; 6 Neurogenetics Research Program, Center for Human Genetics Research, Massachusetts General HospitalBoston, Massachusetts; 7 Center for Pediatric Research, Nemours Biomedical Research, Nemours Alfred I. duPont Hospital for ChildrenWilmington, Delaware; 8 Department of Pediatrics, Thomas Jefferson UniversityPhiladelphia, Pennsylvania; 9 Division of Orthopedics, Nemours Alfred I. duPont Hospital for ChildrenWilmington, Delaware; 10 Division of Neurology, Nemours Alfred I. duPont Hospital for ChildrenWilmington, Delaware

**Keywords:** Array digital PCR, copy number, copy number variation, *SMN1*, *SMN2*, spinal muscular atrophy

## Abstract

Proximal spinal muscular atrophy (SMA) is an early-onset motor neuron disease characterized by loss of *α*-motor neurons and associated muscle atrophy. SMA is caused by deletion or other disabling mutation of *survival motor neuron 1* (*SMN1*). In the human genome, a large duplication of the SMN-containing region gives rise to a second copy of this gene (*SMN2*) that is distinguishable by a single nucleotide change in exon 7. Within the SMA population, there is substantial variation in *SMN2* copy number; in general, those individuals with SMA who have a high *SMN2* copy number have a milder disease. Because *SMN2* functions as a disease modifier, its accurate copy number determination may have clinical relevance. In this study, we describe the development of an assay to assess *SMN1* and *SMN2* copy numbers in DNA samples using an array-based digital PCR (dPCR) system. This dPCR assay can accurately and reliably measure the number of *SMN1* and *SMN2* copies in DNA samples. In a cohort of SMA patient-derived cell lines, the assay confirmed a strong inverse correlation between *SMN2* copy number and disease severity. Array dPCR is a practical technique to determine, accurately and reliably, *SMN1* and *SMN2* copy numbers from SMA samples.

## Introduction

Spinal muscular atrophy (SMA; OMIM #253300) is an early-onset neurodegenerative disease characterized by the loss of *α*-motor neurons (MNs) in the anterior horn of the spinal cord (Crawford and Pardo, 1996). This loss of *α*-MNs is associated with muscle weakness and atrophy. SMA is an autosomal recessive disease and is a leading genetic cause of infant death worldwide with an incidence of 1 in 6000–10,000 births (Pearn, 1978; Cuscó et al., 2002). The carrier frequency for SMA is 1:25–50 in most populations (Ben-Shachar et al., 2011; Su et al., 2011; Sugarman et al., 2012; Lyahyai et al., 2012) though it is lower for some ethnicities (Zaldívar et al., 2005; Labrum et al., 2007; Hendrickson et al., 2009; Sangaré et al., 2014). SMA results from the loss or mutation of *SMN1* (*survival motor neuron 1*; OMIM #600354) on chromosome 5q13 (Lefebvre et al., 1995). In humans, a large tandem chromosomal duplication has lead to a second *SMN2* copy of the gene (OMIM #601627). *SMN2* can be distinguished from *SMN1* by a single-nucleotide difference (c.850C>T) at the outset of exon 7 that disrupts an exonic splice enhancer (Lorson et al., 1999; Monani et al., 1999). As a result, most of *SMN2* mRNAs (about 80–90%) lack exon 7 (*SMNΔ7*) and produce a protein that is both unstable and less than fully functional (Lorson and Androphy, 2000; Burnett et al., 2009). With just 10–20% of the *SMN2* gene product full length and functional, increasing number of *SMN2* partially complements loss of *SMN1* with diminished severity of the phenotype (Coovert et al., 1997; Lefebvre et al., 1997; McAndrew et al., 1997; Prior et al., 2005; Swoboda et al., 2005; Wirth et al., 2006; Tiziano et al., 2007; Elsheikh et al., 2009). The capacity of *SMN2* copy number to modulate phenotype has been extended to transgenic mouse models (Monani et al., 2000; Hsieh-Li et al., 2000; Michaud et al., 2010).

Because *SMN2* copy number influences disease severity in SMA, there is prognostic value in accurate measurement of *SMN2* copy number from patients being evaluated for SMA. Molecular diagnosis of SMA—that is, loss of *SMN1*—has historically been made using a polymerase chain reaction (PCR)-based assay followed by digestion of the PCR product with specific restriction endonucleases (Lefebvre et al., 1995; van der Steege et al., 1995). Numerous assays have been developed to quantify *SMN2* copy number in DNA samples from SMA patients. These assays include radioactive PCR (Coovert et al., 1997), quantitative—or real-time PCR (qPCR)––(Feldkötter et al., 2002; Anhuf et al., 2003; Gómez-Curet et al., 2007), competitive PCR/primer extension (Gérard et al., 2004), denaturing high-performance liquid chromatography (Su et al., 2005), multiplex ligation-dependent probe amplification (Huang et al., 2007), quantitative capillary electrophoresis fragment analysis (QCEFA, Kirwin et al., 2013) and short-amplicon melt profiling (Dobrowolski et al., 2012). An important limitation of these established PCR-based copy number assays is the requirement for a parallel-run calibration curve to assign a breakpoint necessary that identifies placement of an ordinal *SMN2* value.

Digital PCR (dPCR) offers a means of measuring the abundance of a target molecule quantitatively without the need for a calibration curve. In dPCR, the template DNA is distributed across a large number of partitions by limited dilution (Sykes et al., 1992; Vogelstein and Kinzler, 1999). As a result, some of the partitions will lack the template DNA and, as such, will not amplify the target molecule during PCR. By counting the number of partitions containing the amplified target PCR product (positive partitions) and the number of negative partitions, the absolute abundance of the target molecule can be measured in a sample. There are currently two platforms for dPCR—microfluidics and microdroplet emulsion (Day et al., 2013). Zhong et al. (2011) show in a pilot study that droplet dPCR can be used to measure *SMN2* copy number in a small number (*n* = 4) of SMA samples. We demonstrate here the feasibility of using an array dPCR system containing 20,000 partitions in determining the number of copies of *SMN1* and *SMN2* in DNA samples and show that there is a strong correlation between *SMN2* copy number and SMA disease severity.

## Materials and Methods

### Ethics statement

Fibroblast lines generated at the Nemours/Alfred I. duPont Hospital for Children (N/AIDHC) were established following a protocol approved by the N/AIDHC Institutional Review Board, obtained following written informed consent or assent. The cell lines were de-identified so that no protected health information related to these cell lines is known.

### Cell lines

Fibroblast and lymphoblastoid cell lines (LCLs) were either established at N/AIDHC using standard procedures (Villegas and McPhaul, 2005) or obtained from Johns Hopkins University (T. O. C.; Baltimore, MD), the University of Utah (K. J. S.; Salt Lake City, UT) or a cell line repository. Some of the fibroblast lines established at N/AIDHC were obtained from the Molecular Diagnostics Laboratory, while others were established in the Motor Neuron Diseases Research Laboratory from skin samples obtained from the MDA Neuromuscular Clinic or the Nemours Biobank. The following cell lines were obtained from Coriell Cell Repositories (Camden, NJ): GM00232, GM00409, GM00489, GM03813, GM03814, GM03815, GM09677, GM10684, GM22592, GM23255, GM23603, GM23686, GM23687, GM23688 and GM23689. Fibroblast lines UMB-1897, UMB-4648 and UMB-4994 were obtained from the NICHD Brain and Tissue Bank for Developmental Disorders at the University of Maryland (Baltimore, MD). The number of cell lines obtained from non-SMA and SMA patients with varying degrees of disease severity is shown in Table1.

**Table 1:** Clinical information related to the cell lines used in this study

	SMA I	SMA II	SMA III	Unknown SMA	Non-SMA
Cell lines					
Fibroblasts	24	25	10	0	34
LCLs	0	0	1	3	3
Total	24	25	11	3	37
Sex					
Male	14	16	9	2	19
Female	10	9	2	1	18

All fibroblast lines were maintained in Dulbecco’s modified essential medium (DMEM; Life Technologies, Grand Island, NY) containing 10% fetal bovine serum (FBS; Atlas Biologicals, Fort Collins, CO), 2 mmol/L l-glutamine (Life Technologies) and 1% penicillin/streptomycin (Life Technologies). All LCLs were maintained in RPMI-1640 (Life Technologies) containing 15% FBS, 2 mmol/L l-glutamine and 1% penicillin/streptomycin.

### Genomic DNA isolation

Genomic DNA (gDNA) was isolated from fibroblast and LCL cell pellets, using the Gentra Puregene Cell Kit (QIAGEN, Germantown, MD). The pellets were disrupted in 500–1500 *μ*L cell lysis solution depending on the pellet size. The cells were lysed overnight at room temperature in a 15-mL conical tube and then divided in 500-*μ*L aliquots. RNase A Solution (2.5 *μ*L) was added to each aliquot and incubated for 5–60 min at 37°C water bath for five minutes. After chilling the digested aliquots on ice, 165 *μ*L Protein Precipitation Solution was added to each aliquot, vortexed vigorously for 20 sec and then centrifuged for 1 min at 16,000 *g*. The supernatant was then transferred to a clean microcentrifuge tube containing 500 *μ*L 2-propanol. The samples were inverted 50 times to facilitate precipitation of DNA and then centrifuged for 1 min at 16,000 *g*. After the supernatant was discarded, the DNA pellet was washed with 500 *μ*L 70% ethanol and centrifuged for 1 min at 16,000 *g*. The DNA pellets were allowed to air dry for 10 min. After drying, they were re-suspended in 25 *μ*L DNA Hydration Solution and incubated for 60 min at 65°C overnight at room temperature on an orbital shaker. The concentration of the purified gDNA was determined by an ND-2000C NanoDrop spectrophotometer (Thermo Fisher Scientific, Waltham, MA). The integrity of the gDNA was verified by agarose gel electrophoresis.

### Primers and probes

For *SMN1* and *SMN2* copy number determination, the following primers were used (Anhuf et al., 2003): SMN1-Ex7-261R (5′-CCTTAATTTAAGGAATGTGAGCACC-3′) and SMN1-Ex7-116F (5′-AATGCTTTTTAACATCCATATAAAGCT-3′). 6-Carboxyfluorescein (6FAM)-labeled probe oligonucleotides with minor groove binder non-fluorescent quenchers (MGBNFQ) were used to measure *SMN1* [SMN1-Ex7-206T (5′-6FAM-CAGGGTTTCAGACAAA-MGBNFQ-3′)] or *SMN2* [SMN2-Ex7-anti (5′-6FAM-TGATTTTGTCTAAAACCC-MGBNFQ-3′)] signals (the underlined nucleotides are specific for *SMN1* or *SMN2*) (Anhuf et al., 2003). These primers were synthesized by Life Technologies. The primers and 4,7,2′-trichloro-7′-phenyl-6-carboxyfluorescein (VIC)-labeled probe for *RNase P* (*RPPH1*; OMIM #608513) were obtained from the TaqMan™ Copy Number Reference Assay RNase P (Life Technologies). [Corrections added on 13 April 2015, after first online publication: ‘6-Carboxyfluorescein (6FAM)-labeled probe oligonucleotides were used to measure *SMN1* [SMN1-Ex7-206T (5′-6FAM-CAGGGTTTCAGACAAA-3′)] or *SMN2* [SMN2-Ex7-anti (5′-6FAM-TGATTTTGTCTAAAACCC-3′)] signals (the underlined nucleotides are specific for *SMN1* or *SMN2*) (Anhuf et al., 2003)’ has been corrected to ‘6-Carboxyfluorescein (6FAM)-labeled probe oligonucleotides with minor groove binder non-fluorescent quenchers (MGBNFQ) were used to measure *SMN1* [SMN1-Ex7-206T (5′-6FAM-CAGGGTTTCAGACAAA-MGBNFQ-3′)] or *SMN2* [SMN2-Ex7-anti (5′-6FAMTGATTTTGTCTAAAACCC-MGBNFQ-3′)] signals (the underlined nucleotides are specific for *SMN1* or *SMN2*) (Anhuf et al., 2003)’.]

For Sanger sequencing of SMN, the following primers were used: SMN-SEQ7F (5′-CAAAATGCTTTTTAACATCCATATAA-3′) (Vezain et al., 2010), SMN-SEQ7R (5′-AAACATTTGTTTTCCACAAACC-3′) (Vezain et al., 2010), SMNaf (5′-TGCGCATCCGCGGGTTTGCT-3′) (Parsons et al., 1998) and SMNcr (5′-TCATTTAGTGCTGCTCTATGCCA-3′) (Parsons et al., 1998). These sequencing primers were synthesized by Integrated DNA Technologies, Inc. (Coralville, IA).

### SMN sequencing

To determine if each sample contained *SMN1* and/or *SMN2*, exon 7 of SMN was amplified by PCR, using SMN-SEQ7F and SMN-SEQ7R as primers (Vezain et al., 2010). The PCR conditions were as follows: 5 min 96°C followed by 40 cycles of 30 sec 96°C, 30 sec 56°C and 1 min 72°C followed by a final extension step for 7 min at 72°C. The PCR product was purified using the Wizard SV Gel and PCR Cleanup System (Promega, Madison, WI) according to manufacturer’s directions. The purified PCR product was sequenced with the ABI 3130xl Genetic Analyzer (Life Technologies) automated sequencer, using the BigDye Terminator v3.1 Cycle Sequencing kit (Life Technologies).

### SMN1 and SMN2 copy number assays

**Figure 1:**
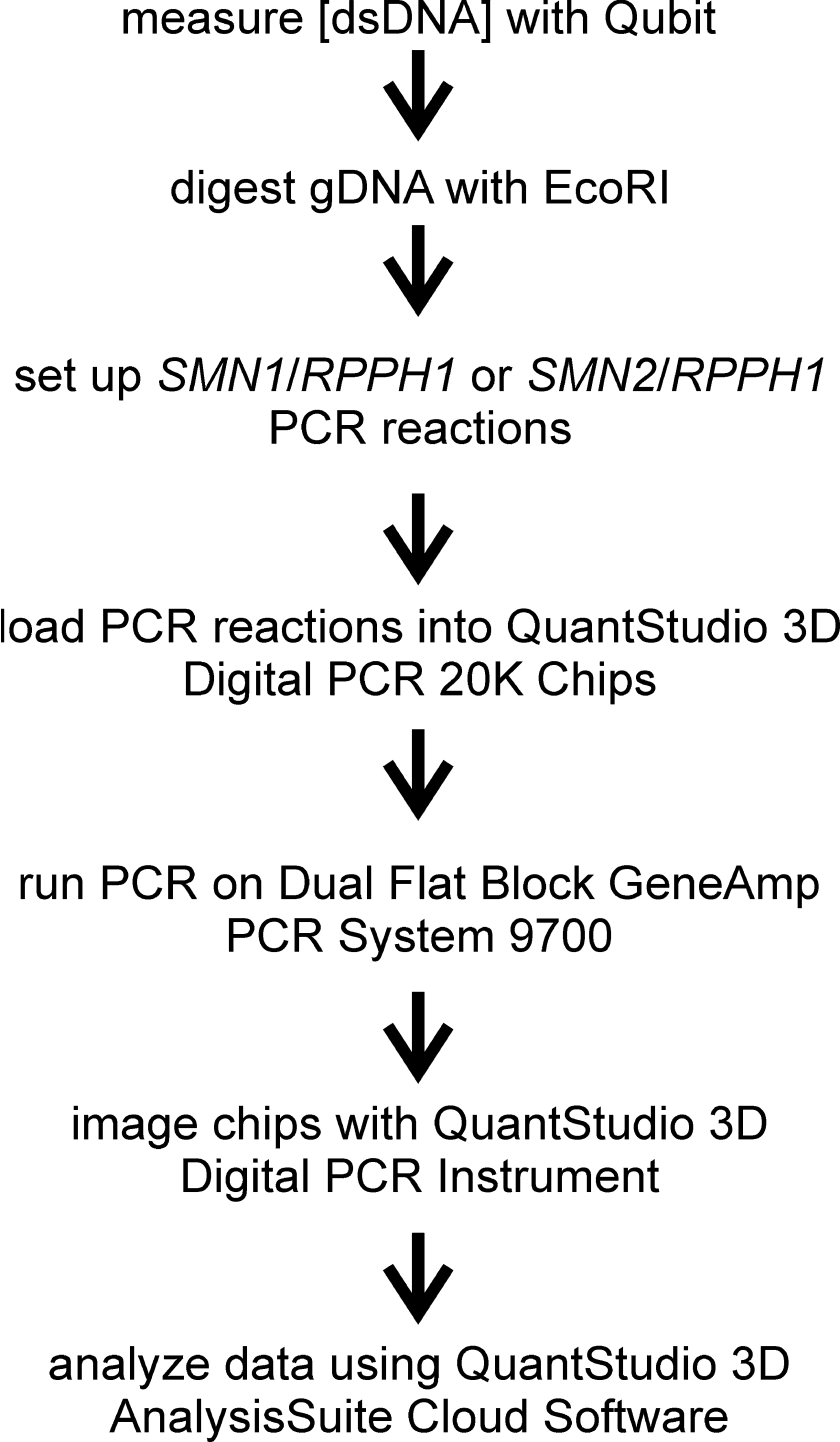
Workflow for *SMN1*/*SMN2* copy number assays, using the QuantStudio 3D array dPCR system.

The copy numbers of *SMN1* and *SMN2* were measured in each gDNA sample using the QuantStudio™ 3D Digital PCR System (Life Technologies). Figure1 shows the workflow of the *SMN1*/*SMN2* copy number assays. The concentration of double-stranded DNA (dsDNA) was measured from each sample using the Qubit™ dsDNA Broad Range Assay kit (Life Technologies). 400 ng dsDNA was digested with 20 U *Eco*RI (New England Biolabs, Inc., Ipswitch, MA) for 60 min at 37°C. After thermal denaturation (20 min at 65°C), the digest DNA was diluted fourfold with nuclease-free ddH_2_O.

*RPPH1* and either *SMN1* or *SMN2* signals were amplified in each PCR reaction. Each PCR reaction contains 30–60 ng *Eco*RI-digested gDNA, *RPPH1* primer/probe mix (1×), *SMN1*/*SMN2* primer probe mix (900 nmol/L SMN1-Ex7-116F, 900 nmol/L SMN1-Ex7-261R, 200 nmol/L either SMN1-Ex7-206T or SMN2-Ex7-anti) and QuantStudio™ 3D Digital PCR Master Mix. Each PCR reaction was then loaded into a QuantStudio™ 3D Digital PCR 20K chip according to manufacturer’s directions. The chips were then loaded onto the Dual Flat Block GeneAmp™ PCR System 7900 and PCR was performed using the following conditions: 10 min at 96°C followed by 39 cycles of 2 min at 60°C, and 30 sec at 98°C followed by 2 min at 60°C. 6FAM and VIC images from each chip were then taken with the QuantStudio™ 3D Instrument which provided the copies of *SMN1* (or *SMN2*)/*μ*L (6FAM) and of *RPPH1*/*μ*L (VIC). The raw data were subsequently analyzed using the QuantStudio™ 3D AnalysisSuite Cloud Software. The copy number of *SMN1* or *SMN2* was calculated with the following equation:

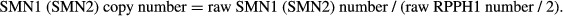


### Statistical analysis

The coefficient of variation (CV) was used to measure the reliability of the QuantStudio™ 3D dPCR assay (Gómez-Curet et al., 2007). The CV is defined as the standard deviation (SD) of the copy number divided by the mean copy number for all subjects with the same copy number. Spearman’s correlation analysis (*r*) was used to determine the relationship between *SMN2* copy number and disease severity in the SMA samples. All statistical analyses were performed with SPSS v.22.0 (IBM, Armonk, NY).

## Results

We measured the number of *SMN1* and *SMN2* copies in gDNAs isolated from cell lines derived from SMA patients as well as from healthy non-SMA subjects using array dPCR (Fig.1). *SMN1* or *SMN2* dPCR was multiplexed with *RPPH1* because the copy number of *RPPH1* does not vary amongst the human population (Baer et al., 1990). The gDNA templates were digested with *Eco*RI prior to PCR amplification as there are no *Eco*RI restriction sites within the *SMN1*, *SMN2* or *RPPH1* regions of amplification. The array dPCR assay detected accurately and reproducibly from 0 to 3 copies of *SMN1* and 0 to 5 copies of *SMN2* in the analyzed samples.

The assay conditions were first tested on *SMN1* and *SMN2* reference standards (*n* = 7) obtained from the Clinical Laboratory Improvement Amendments (CLIA)-certified Molecular Diagnostics Laboratory at N/AIDHC. These standards were generated from genomic DNA extracted from blood specimens, with copy numbers assessed by QCEFA (Kirwin et al., 2013). One of these reference samples (SDC1) could not be accurately accessed for *SMN2* copy number using QCEFA. Blinded array dPCR determination of *SMN1* and *SMN2* copy numbers in the reference samples matched that obtained by QCEFA (Table2). Of interest, sample SDC1 measurements fell well within the detection capability of array dPCR and this sample carried 4 copies of *SMN2*.

**Table 2:** Comparison of *SMN1* and *SMN2* copy numbers measured by quantitative capillary electrophoresis fragment analysis (QCEFA) and by array dPCR

Sample	QCEFA		Array dPCR	
	*SMN1*	*SMN2*	*SMN1*	*SMN2*
SDC1	0	≥4	0	4
SDC2	1	3	1	3
SDC3	2	1	2	1
SDC4	2	2	2	2
SDC5	1	1	1	1
SDC6	0	3	0	3
SDC7	1	1	1	1

**Figure 2:**
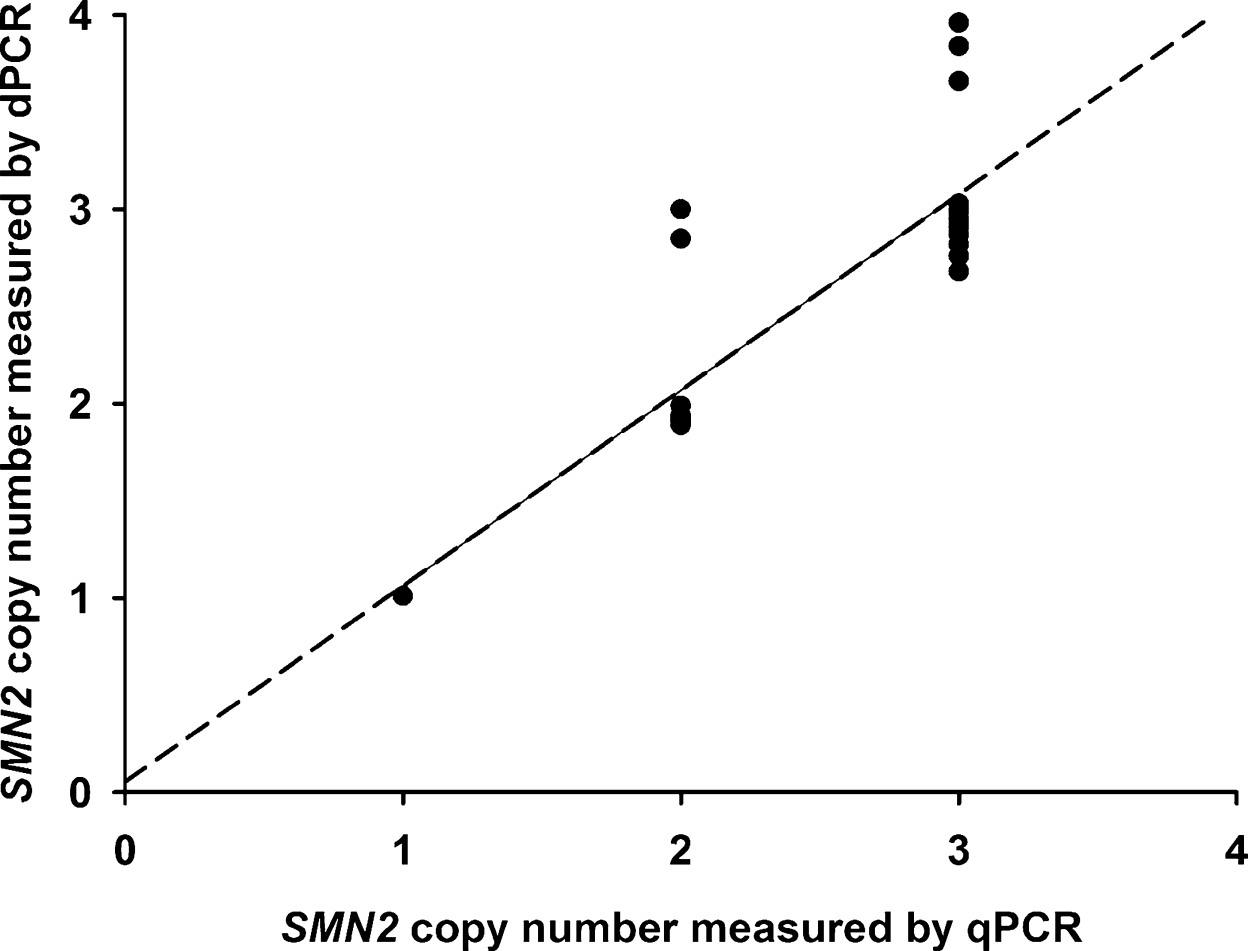
Comparison of *SMN2* copy number in SMA samples determined by qPCR to that by array dPCR. The dashed line represents the linear relationship between *SMN2* copy number determined by TaqMan™ qPCR (Gómez-Curet et al., 2007) and that determined by array dPCR.

This validation phase also included a comparison between array dPCR and real-time TaqMan™ qPCR (qPCR). The *SMN2* copy numbers for a subset of our SMA samples (*n* = 30) were determined previously (Gómez-Curet et al., 2007). We saw good concordance between the 2 techniques for samples carrying low *SMN2* copy number (i.e., ≤2 copies); however, the concordance dropped to 80% (12/15) for samples carrying higher *SMN2* copy numbers (Fig.2). This result highlights the inability of TaqMan™ qPCR to accurately measure copy numbers greated than 3 (Gómez-Curet et al., 2007; Prior et al., 2011). To test the upper limit of detection for this new assay, we used gDNA from a set of 4 SMA (GM00232, GM03813, GM09677, and GM10684) and 1 carrier (GM03814) Coriell Cell Repositories cell lines that were shown to contain 2, 3, 3, 2, and 5 *SMN2* copies, respectively, using droplet dPCR (Zhong et al., 2011). Our copy number measurements were in complete concordance with the published droplet dPCR results, most notably for GM03814 which carried a high copy number of *SMN2*.

To determine the reliability of the array dPCR copy number assays, the mean CV was calculated for each *SMN1* and *SMN2* copy number measurement for both the SMA (Table3) and non-SMA (Table4) samples derived from cell lines (Gómez-Curet et al., 2007). In both SMA and non-SMA samples, the CVs for each *SMN1* and *SMN2* copy numbers were below 4% demonstrating that our array dPCR copy number assays were reliable.

**Table 3:** Coefficient of variation (CV) measurements for *SMN2* copy numbers in SMA patient samples

SMA phenotypic grade	Expected copy number	Measured copy number (mean ± SD)	CV
Type I	2	1.939 ± 0.046	0.024
	3	2.925 ± 0.106	0.036
Type II	1	1.180	–
	2	1.930	0.000
	3	2.891 ± 0.106	0.037
	4	3.860	–
Type III	1	1.010	–
	3	2.923 ± 0.061	0.021
	4	3.884 ± 0.102	0.026
Unknown	2	1.900 ± 0.071	0.037
	3	2.820	–

**Table 4:** Coefficient of variation (CV) measurements for *SMN1* and *SMN2* copy numbers in non-SMA samples

Gene	Expected copy number	Measured copy number (mean ± SD)	CV
*SMN1*	1	0.972 ± 0.016	0.017
	2	1.926 ± 0.053	0.027
	3	2.905 ± 0.069	0.024
*SMN2*	0	0.00	–
	1	0.991 ± 0.028	0.032
	2	1.930 ± 0.072	0.037
	3	2.840 ± 0.046	0.016
	5	4.72	–

**Figure 3:**
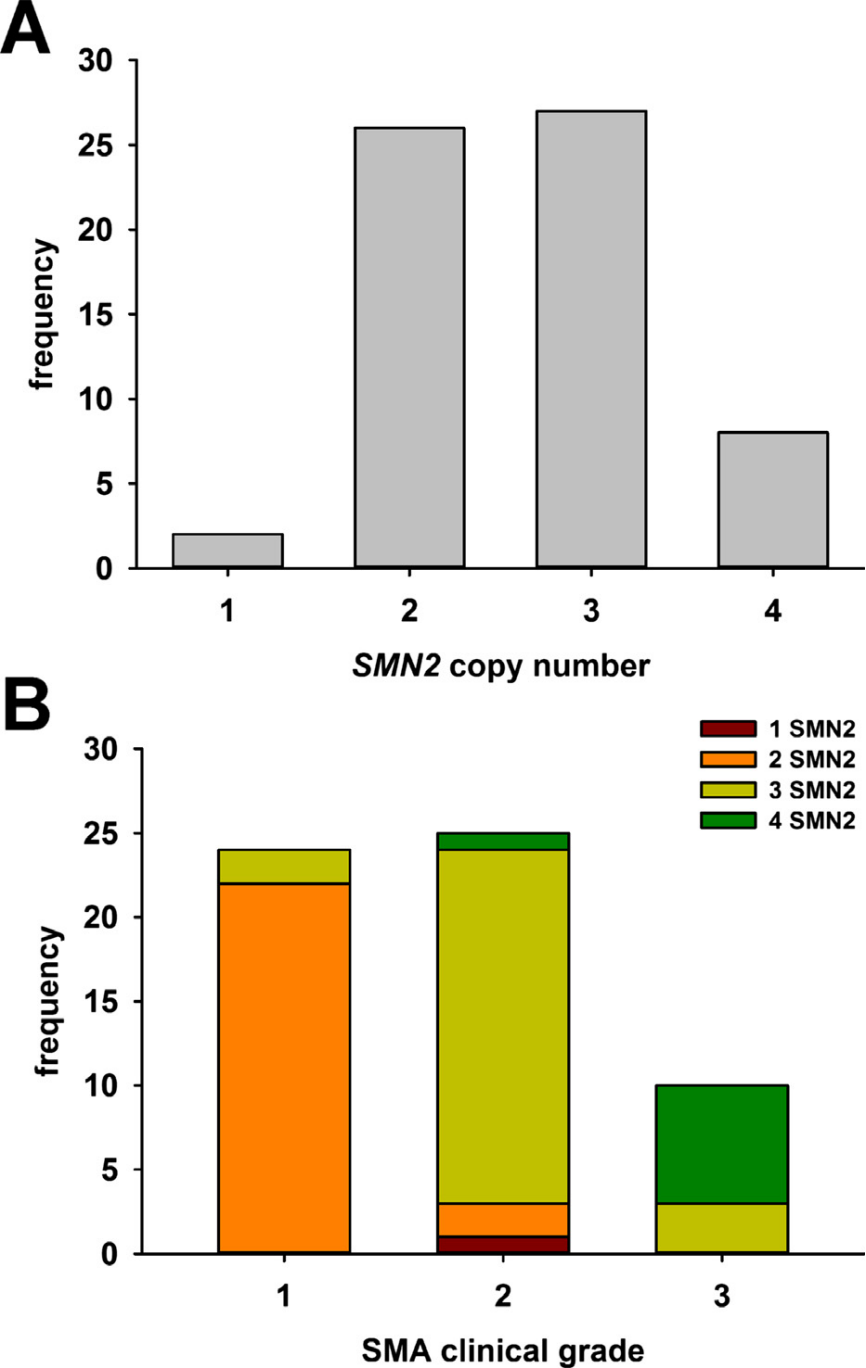
*SMN2* copy number in SMA samples. (A) Distribution of *SMN2* copy number in the SMA patient samples (*n* = 60). (B) Relationship between *SMN2* copy number and disease severity in *SMN1*-deleted SMA samples (*n* = 59). Each bar represents a clinical grade of SMA. The distribution of *SMN2* copy numbers (1 *SMN2*, red; 2 *SMN2*, orange; 3 *SMN2*, yellow and 4 *SMN2*, green) within each clinical grade is shown within each bar.

Using these assay conditions, we determined the copy numbers of *SMN1* and *SMN2* for all of the cell lines within our collection. Our collection contained both fibroblasts and Epstein–Barr virus (EBV) immortalized LCLs. One hundred cell lines—63 of which were derived from SMA patients—were used in this study (Table1). All but one of the 63 SMA DNA samples had a loss of both *SMN1* alleles as determined by using Sanger sequencing. The remaining cell line harbored one deletion allele and one missense mutation in *SMN1* (*c.38C>G*; SMN1p.A2G). Most of the SMA samples contained 2 or 3 copies of *SMN2* (Fig.3A). For those SMA patients harboring deletions of *SMN1* and whose disease severities were known (*n* = 59), patients with higher *SMN2* copy numbers (i.e., 3 or 4) had milder disease severities than those patients with fewer copies of *SMN2* (i.e., 2; Fig.3B). In fact, there was a strong, negative correlation between *SMN2* copy number and SMA disease severity within this group (*r* = 0.830, *P* < 0.001).

**Figure 4:**
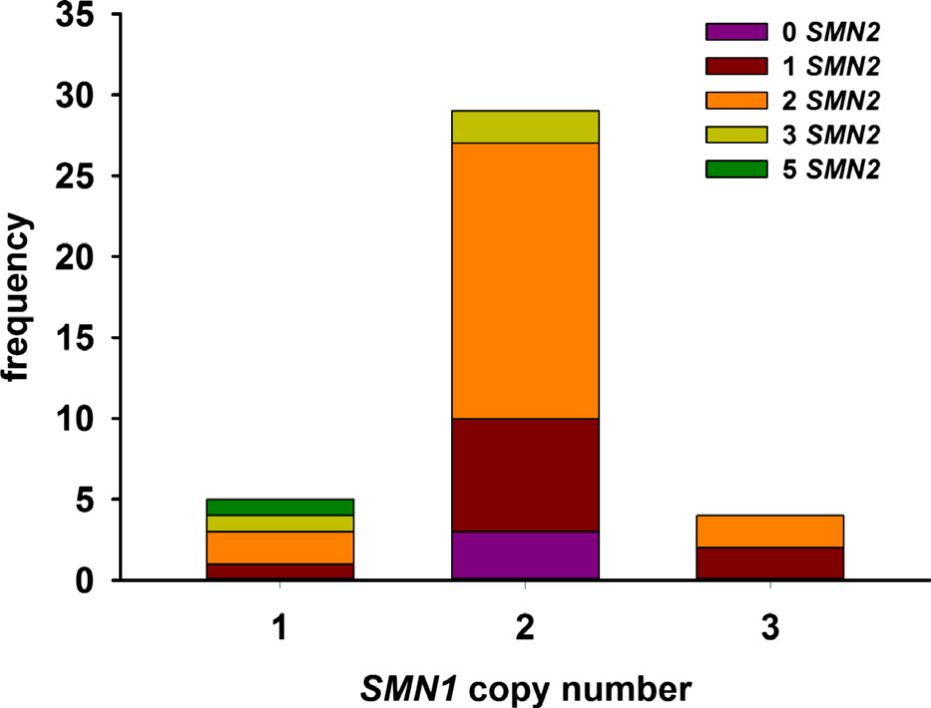
*SMN1* and *SMN2* copy numbers in non-SMA samples. Each bar represents a copy number for *SMN1* in the cohort of non-SMA samples (*n* = 40). The distribution of *SMN2* copy numbers (0 *SMN2*, purple; 1 *SMN2*, red; 2 *SMN2*, orange; 3 *SMN2*, yellow and 5 *SMN2*, green) within each *SMN1* copy number is shown within each bar. None of the samples in our cohort contained 4 copies of *SMN2*.

In the non-SMA samples, we found variation in both *SMN1* and *SMN2* copy numbers (1–3 copies for *SMN1* and 0–5 copies for *SMN2*; Fig.4). The most common *SMN1*:*SMN2* copy number combination observed within our population was 2:2; this combination has been observed in other studies as well (Anhuf et al., 2003; Gérard et al., 2004; Pyatt and Prior, 2006; Gómez-Curet et al., 2007).

## Discussion

We established new array dPCR *SMN1* and *SMN2* copy number assays that accurately measured copy numbers in SMA as well as in non-SMA DNA samples isolated from whole blood cells and cell lines derived from fibroblasts and lymphoblasts. The dPCR-derived *SMN1* and *SMN2* copy numbers matched those found in reference standards used by a diagnostic laboratory and in a limited number of cases using microdroplet dPCR (Zhong et al., 2011). *SMN2* copy numbers in SMA DNA samples were concordant with those results measured by TaqMan™ qPCR (Gómez-Curet et al., 2007) at low *SMN2* copy numbers but the concordance was not as strong at higher (i.e., >3) *SMN2* copy numbers. The majority of dPCR / TaqMan™ qPCR mismatches occurred at higher *SMN2* copy numbers where the TaqMan™ qPCR assay cannot easily distinguish unit differences (Gómez-Curet et al., 2007; Prior et al., 2011). Array dPCR detected unit differences in *SMN2* copy number over a wide range of *SMN2* copy numbers similar to droplet dPCR (Zhong et al., 2011). Because of this wide range of detection, dPCR can be very useful in accurately quantifying *SMN2* copy number in patients with milder forms of SMA, that is, type III SMA, who generally have higher *SMN2* copy numbers.

The reliability of the array dPCR assays was determined by comparing the coefficients of variation (CV) for all samples with the same copy number. Our array dPCR results had a 1.6–3.7% CV for *SMN1* and 2.1–3.7% CV for *SMN2*. In contrast, the TaqMan™ qPCR assay shows a 5.2–9.7% CV for *SMN1* and a 0.8–7.6% CV for *SMN2* (Gómez-Curet et al., 2007). The greater reliability of the array dPCR assays when compared against the TaqMan™ qPCR assays is a result of the random distribution of template DNA molecules within the 20,000 partitions in microfluidic dPCR array (Whale et al., 2014).

Using array dPCR, we have confirmed a very strong inverse correlation between *SMN2* copy number and disease severity in our SMA patient samples. Numerous previous studies also document a similar relationship between *SMN2* copy number and SMA disease severity (Coovert et al., 1997; Lefebvre et al., 1997; McAndrew et al., 1997; Prior et al., 2005; Swoboda et al., 2005; Wirth et al., 2006; Tiziano et al., 2007; Elsheikh et al., 2009; Crawford et al., 2012). *SMN2* copy number is associated with many measures of SMA phenotype severity including gross motor function, forced vital capacity, muscle mass, and denervation (Swoboda et al., 2005; Rudnik-Schöneborn et al., 2009; Kaufmann et al., 2011; Crawford et al., 2012; Kaufmann et al., 2012). Many current and future clinical trials for SMA will use these outcomes measures along with changes in SMN expression to gauge efficacy (Nurputra et al., 2013). Because *SMN2* copy number is a defining criteria of eligibility to many SMA clinical trials, accurate and reliable measurements will continue to be essential to clinical research.

In some cases within our pool of SMA samples, there were SMA patients with low *SMN2* copy numbers exhibiting a milder phenotype. A rare variant in *SMN2*, *SMN2 c.859G>C*, may explain this finding as it results in a partial rescue of the truncated, exon 7 excluded, transcript that characterized most of the mRNA generated from *SMN2* (Prior et al., 2009; Vezain et al., 2010). Array dPCR will aid in the identification of cases having mismatches from the expected genotype–phenotype relationship. Identifying such mismatches could lead to the identification of potential complementing mutations in *SMN2* like *SMN2 c.859G>C*.

Array dPCR can be easily used to measure *SMN1* and *SMN2* copy numbers accurately in DNA samples obtained from SMA patients and healthy, non-SMA controls. Array dPCR can accurately determine copy number within a wider range of *SMN2* copies (0 to at least 5 copies) than either qPCR (0–3 copies) or QCEFA (0–4 copies). Unlike qPCR, dPCR does not require a calibration curve to assign a numeric measure of copy number (Day et al., 2013). Because the template DNA molecules are randomly distributed amongst the 20,000 partitions in dPCR, copy number measurements are more precise and reliable when compared against qPCR (Whale et al., 2014). For these reasons, array dPCR has advantages in comparison to conventional diagnostic measurements of *SMN1* and *SMN2* copy number in SMA patient DNA samples. Future work using a larger cohort of gDNA extracted from blood samples will determine the applicability of array dPCR for SMA diagnostics and as a prognostic tool.
